# The Role of Zinc and Junctional Adhesion Molecule A Protein in Platelet Plug Formation

**DOI:** 10.3390/medicines13040028

**Published:** 2026-09-29

**Authors:** Sai Harsha Nagidi, Jonah Stringham, Ethan Firth, Brent Lisonbee, Chris Hart, Dario Mizrachi

**Affiliations:** 1 Arizona College of Osteopathic Medicine (AZCOM), Midwestern University, Glendale, AZ 85308, USA; nagidiharsha@gmail.com; 2 Department of Cell Biology and Physiology, Brigham Young University, Provo, UT 84604, USA; 3 Department of Biomedical Sciences, Noorda College of Osteopathic Medicine, Provo, UT 84606, USA; do29.etfirth@noordacom.org (E.F.); do29.bllisonbee@noordacom.org (B.L.); do29.cjhart@noordacom.org (C.H.)

**Keywords:** primary hemostasis, zinc, platelet plug, platelet degranulation

## Abstract

**Background**: The coagulation cascade depends on the active participation of several elements present in the blood as well as signals arising from the endothelial cells. A platelet plug is a temporary, fast-response seal formed by platelets at the site of a damaged blood vessel to initiate hemostasis. Among the changes platelets undergo is the degranulation step. Platelet degranulation results in the release of substances like ADP, serotonin, fibrinogen, and zinc. Currently, zinc’s targets are unknown. **Methods**: Platelet-to-platelet aggregation relies on specific surface receptors and plasma bridge proteins. Such mechanistic elements have been extensively studied (i.e., αIIbβ3 integrin). In this work, using platelet aggregation methods and recombinant proteins, we will expand the repertoire of proteins relevant to platelet plug formation. Particularly, size-exclusion chromatography (SEC), surface plasmon resonance (SPR), and transmission electron microscopy (TEM) were mainly used. Using 1 mM zinc and 10 µM ADP, we evaluated platelet aggregation and proteins responsible for this aggregation’s behavior in response to zinc release from platelets’ granules upon activation. SPR provided results to evaluate the affinity of our target proteins on the surface of platelets when influenced by the increase in zinc after degranulation. **Results**: In the present work, we provide evidence that the high local concentration of zinc is intended to target junctional adhesion molecule A (JAM-A), which remains inactive (inhibiting cell-adhesion and cytoskeleton dynamics) when coagulation is not needed, and platelets move through the bloodstream as single units. Zinc-activated JAM-A leads the platelets to aggregate. Our experimentation includes work with platelets and a synthetic biology small peptide to quench the effects of zinc. **Conclusions**: JAM-A interacts with zinc to aid in the formation of the platelet plug. This interaction activates the cell-adhesion and cytoskeleton dynamics functions of JAM-A. We suggest that further exploring this mechanism of zinc-activated JAM-A can be advantageous for understanding hemostasis, its role in antithrombotic therapy, coagulation inhibition, or thrombosis prevention.

## 1. Introduction

Hemostasis is the process that aims to prevent blood loss due to trauma or injury that affects the vasculature. It begins with the rapid formation of a platelet plug at a site of vascular injury, driven by platelets interacting with exposed subendothelial structures and adhesive proteins like von Willebrand factor (vWF) [1]. The result of primary hemostasis is the formation of a stable platelet plug around which the fibrin network is built [2]. This same process is responsible for pathogenic thrombus formation. Disorders of primary hemostasis tend to manifest as mucosal bleeding, including epistaxis, oral bleeding, and menorrhagia, and often immediate difficulty with hemostasis in the post-operative setting [3].

Platelet aggregation is recognized as critical for hemostatic platelet plug formation. Until recently, platelet aggregation was considered a well-characterized process involving the noncovalent bridging of integrin αIIbβ3 receptors on the platelet surface by the dimeric adhesive protein fibrinogen [4]. Nevertheless, more recently it has become clear that this process is more complex and dynamic. A consequence of this realization is a public website (PlateletWeb) dedicated to platelets [5].

During platelet plug formation, platelets transition from a single platelet state to a multi-platelet aggregate. As such, the platelet plug forms contact with other proteins or factors, or with other platelets. Platelets possess numerous cell-adhesion proteins on their surface, primarily glycoproteins (GPs) and integrins, that are crucial for hemostasis, thrombosis, and interactions with other cells [6]. These receptors bind to collagen or extracellular matrix proteins upon injury, allowing platelets to adhere, activate, and aggregate [6].

In addition to GPs and integrins, platelets contain junctional adhesion molecules A and C (JAM-A and JAM-C) [7]. JAMs belong to the immunoglobulin domain superfamily (IgSF), one of the largest protein families, with over 700 members [8]. Key structural features of immunoglobulin domains are a beta-sandwich fold made of 7–9 antiparallel beta strands, usually arranged in two beta sheets with a Greek-key topology. They are typically ~70–130 amino acids long depending on whether they are constant or variable domains, and a conserved intradomain disulfide bond helps stabilize the fold [9,10]. JAMs have similar characteristics, typically two Ig domains, D1 and D2, with the distal domain engaging in JAM-JAM interactions [11]. JAM proteins are key components of Tight Junctions in endothelium and epithelium [12].

It is intriguing to understand the role of JAM-A and JAM-C as cell-adhesion molecules. Prior to primary hemostasis activation, these proteins remain inactive, as platelets persist singly in the bloodstream. The literature offers another puzzling detail about the effects of JAM-A on the surface of platelets. Sobocka and colleagues [13], described the activation of JAM-A on the surface of platelets by using an antibody targeting JAM-A. Among the consequences of anti-JAM-A binding to JAM-A on the surface of the platelets, a star-shaped morphology is acquired by platelets [13]. Sobocka and colleagues [13] suggested that the antibody potentiated the agonist-induced platelet aggregation’s shape in the absence of platelet aggregation since JAM-A was blocked by the antibody from forming homotypic interactions. The antibody seemed to overcome the threshold of activation of JAM-A to the point that its intracellular domain interacted with adapter proteins that in turn interact with the actin cytoskeleton [14]. JAM-A is considered inactive prior to hemostasis, and when this event is initiated, JAM-A becomes a key participant in the platelet plug by enabling the platelet’s shape to be altered to a star shape, increasing surface area for platelet–platelet contact. Thus, JAM-A signals intracellularly to the cytoskeleton and extracellularly to act as a cell-adhesion molecule.

Another intriguing aspect of platelet activation is the degranulation steps, which, among other molecules, release Zn^2+^. Zinc is a critical regulator of platelet function, stored in α-granules and released during platelet degranulation to amplify platelet activation, fibrin formation, and thrombus growth [15]. It acts as a signaling molecule, where increased local extracellular Zn^2+^ enhances activation, and it acts as a potent agonist during injury [15]. Zinc deficiency hinders hemostasis, leading to reduced aggregation, impaired platelet function, and increased bleeding, while abnormal zinc levels can alter platelet reactivity through calcium-dependent signaling. Gray platelet syndrome (GPS) is a rare inherited disorder caused by NBEAL2 gene mutations that lead to defective α-granule biogenesis [16]. It is characterized by macrothrombocytopenia (large, low-count platelets), bleeding, bone marrow fibrosis, and an “empty” gray appearance on blood smears due to lack of α-granules [17]. In GPS, loss of these granules impairs platelet function, so patients typically have easy bruising, nosebleeds, prolonged bleeding after procedures, and sometimes heavy menstrual bleeding [18]. Zinc levels in plasma range between 10 and 20 µM, but free/labile zinc is much lower because most is protein-bound [15]. In addition, diagnostic X-ray spectrometry (DXS) has been used to quantify the multiple elemental composition of human platelets; among the ions evaluated, zinc concentrations were measured and found to reach 35 μg/g. These values show that platelets contain significant pools of zinc that are 30–60-fold higher than plasma levels [19]. Thus, during hemostasis zinc levels may reach upwards of 1 mM.

Hemostasis is a complex and dynamic process. We have presented two known events that occur during primary hemostasis: platelet degranulation and activation of JAM proteins that signal intracellularly to change platelet morphology and simultaneously awaken their cell-adhesion function to enhance platelet plug formation.

Our laboratory has studied the JAM protein family in recent years. We have examined how the extracellular domains self-assemble into homodimers and also as heterodimers with other members of their family [20]. We propose that JAM-C homomers possess the strongest binding affinity, only to be surpassed by the heteromeric interactions of JAM-A and JAM-C. More recently, we have suggested that extracellular Calcium can orchestrate the formation of mature Tight Junctions at the cell membrane [21]. Among the membrane proteins that form the Tight Junction (claudins, occludin, and JAMs), JAMs may be the only Calcium-dependent [21]. We presented evidence that the Tight Junction, influenced by Calcium can interact with E-cadherin (a calcium-dependent cell-adhesion molecule [22,23]) to organize the apical junctional complex (AJC) [21]. A follow-up publication demonstrated that the extracellular domain of JAM-A was susceptible to other cations, not just calcium, but zinc, iron (II), iron (III), and magnesium as well, and the presence of these cations increased self-aggregation in JAM-A [24]. In that study, Mendoza and colleagues [24] performed Surface Plasmon Resonance to demonstrate that JAM-A had the strongest self-affinity and number of aggregation molecules in the presence of Zinc.

In this present work, we highlight evidence to support our hypothesis: primary hemostasis activation leads to platelet degranulation, increasing local concentration of Zn^2+^; JAM-A binds Zn^2+^ to enhance platelet plug formation. Our data will suggest the participation of JAM-C in this process, but further experimentation is needed to draw further conclusions. Perhaps studies dedicated to JAM-C. Further understanding the dynamics of hemostasis is highly relevant to intervention in healthcare.

## 2. Materials and Methods

### 2.1. Materials

New England Biolabs (Ipswich, MA, USA) was the provider of bacterial cells. Bacterial cell culture and general salts and buffers were obtained from Genesee Scientific (El Cajon, CA, USA). Control protein Hsp60 6xHis tag (catalog # 230-30126, Ray Biotech, Corners, GA, USA). Antibody, anti-6xHis mouse monoclonal antibody (MA1-135; Invitrogen, Carlsbad, CA, USA).

### 2.2. Plasmids

TWIST Biosciences (San Francisco, CA, USA) was selected as the provider to synthesize DNA and perform cloning. DNA was codon-optimized for K12 *E. coli* prior to ordering its synthesis.

### 2.3. Electron Microscopy

Cells at the end of the experiment were prepared for negative staining by applying 5 μL of cells. Cells were deposited onto a carbon-coated grid (300-mesh copper grid) for 2 min. To complete negative staining, a second solution of 1.5% uranyl acetate was immediately applied for another 2 min. Grids were dried and used immediately using an FEI Tecnai 12 Spirit Twin electron microscope. View fields for each sample were randomly selected and photographed at different magnifications. Image analysis was completed with ImageJ software (Version 1.54r, 25 September 2025).

Freshly isolated platelet-rich plasma (PRP) was prepared for negative staining by applying 5 μL of the sample onto a 300-mesh carbon-coated copper grid for 2 min and blotting with filter paper to remove excess solution. The grid was immediately stained with 1.5% uranyl acetate for 2 min and air-dried before examination under the microscope (same as above for *E. coli)*. Twenty fields per sample were randomly selected, photographed at various magnifications, and analyzed using ImageJ software (Version 1.54r).

### 2.4. Protein Expression and Purification

Our protocols for protein expression and purification have been published previously [25]. Briefly, we employed Terrific broth (TB) medium for cell growth at 37 °C. When OD600 was 0.8–1, IPTG was added to a final concentration of 1 mM. The temperature was brought to 16 °C for a period of 18 h. TWIST Bio prepared all synthetic DNA containing an N-terminal 6xHis tag. This protein tag was used for NiNTA protocols of protein purification [25]. Western blot protocols for protein expression were employed with anti-6xHis monoclonal antibody (MA1-135; Invitrogen, Carlsbad, CA, USA) at a dilution of 1:20,000. Final steps toward developing the image were carried out using the LI-COR system (Lincoln, NE, USA). TATAx3: prepared the iDRIVE-PFP (also known as iDRIVE-TATAx3) protein to be expressed and purified according to our own protocol [26]. Yields of SPEAR were near 10 mg/L.

### 2.5. Surface Plasmon Resonance

SPR experimentation was carried out on the Nicoya OpenSPR machine. Proprietary software helps us calculate the binding affinity between a protein and a ligand. Carboxyl sensor chips are employed due to their negative charge properties. The ligand is prepared so that the pH of the sodium acetate buffers is 0.5–1 units below the isoelectric point, giving the ligand a positive charge. The proteins are immobilized in the Carboxyl sensor chip. Furthermore, the amine groups can form a covalent bond with the carboxyl surface after it is activated by EDC/NHS. Following the blocking step (manufacturer’s buffer), 200 µL of 1 M sodium caprate is administered to disrupt the preformed protein–protein interactions (PPIs). Triplicate injections of the analyte protein are performed at concentrations of 12.5 µg, 25 µg, 50 µg, and 100 µg per 200 µL of injection. Caprate injections (disrupt PPIs between injections) are performed after each analyte interaction is concluded. The close curve fitting to the sensograms is calculated by global fitting curves (1:1 Langmuir binding model). The data are retrieved and analyzed with TraceDraw software 1.10 (Kitchener, ON, Canada).

### 2.6. Size Exclusion (SEC)

Protein in the amount of 0.250 mg is injected into the NGC Chromatography System (ChromeLab software, Bio-Rad, Hercules, CA, USA). A solution of 30 mM HEPES, 100 mM NaCl, with 3 mM Ca^2+^ is used as our control for the proteins. An ENrich SEC 650 column of 109,300 mm, 24 mL, pre-packed high-resolution SEC 650 size-exclusion column, size range 5k–650 kDa (Bio-Rad) was selected for the runs. Analysis of elution volumes is carried out with SEC software (ChromeLab software, Bio-Rad). Standards from BIO-RAD are used to determine the size of the peaks (Protein Standard for Anion Exchange Chromatography, Bio-Rad cat #1250561).

### 2.7. SDS-PAGE

For quality control of purified proteins (e.g., size) and purity of the preparation, we loaded dye containing 1% SDS, 0.125 M Tris (pH 6.8), and 40% glycerol. After preparing our proteins and denaturing them, we ran them on a 10% SDS-PAGE gel. After conducting gel electrophoresis (150 V), protein bands were visualized by staining with Coomassie Brilliant Blue for 2 h. 

### 2.8. Circular Dichroism

CD measurements were performed on the Spectrophotometer Model 420 AVIV (Biomedical Inc., Lakewood, NJ, USA). The far-UV–CD spectra of 0.100 µM target protein were equilibrated with PBS (pH 7.4–8.0) and recorded in a 100 mm QS glass cuvette cell. For the analysis of thermal stability and changes in the secondary structure, 0.100 µM of protein sample was incubated at 4 °C, 22 °C, 37 °C, 50 °C, and 75 °C, and monitored by measuring changes in ellipticity at 260 nm to 195 nm using 20 s scans. Secondary structure percentages were obtained by Bestsel circular dichroism analysis.

### 2.9. Platelet Isolation and Aggregation Assay

Platelet isolation was achieved by centrifugation of blood at 800× *g* for 5 min. To eliminate trace thrombin generation during processing, blood was collected in tubes containing 3.2% sodium citrate supplemented with 40 µM PPACK. This specific inhibitor was maintained throughout the platelet isolation process. Effects of platelet aggregation of PRP in the presence of vehicle (PBS) or PBS-solved JAMAspear can be observed in Results and Discussion Section 3.7. The total aggregation after 20 min of the experiment reached OD_405nm_ values of −0.001. On the other hand, ADP-induced aggregation of PRP reached values of −0.2 (Results and Discussion Section 3.7). A BioTek Synergy H1 plate reader and its software Gen5 (Agilent, Santa Clara, CA, USA) were used to read platelet aggregation at OD_405nm_, in a 96-well plate, with a total volume of 200 µL/well. Platelet-rich plasma (PRP) was prepared according to a protocol published by Dhurat and Sukesh [27]. Reagents were used at a final concentration of 1 mM Zinc sulfate, 10 µM ADP, and 100 µM JAMAspear. Seventy-five microliters of PRP were used per well. Final volume was achieved using PBS. The reaction was monitored for 20 min after mixing, and data were recorded every 20 s. Final plots were prepared with GraphPad Prism 11.0. The plots were constructed from four independent experiments and fitted to nonlinear regression under exponential and one-phase decay. For each experiment, matched platelet-free matrix controls containing vehicle, zinc, ADP, SPEAR, or their combinations were included to assess treatment-dependent background optical signals. Aggregation-associated OD changes were interpreted relative to matched controls and were evaluated in conjunction with flow-cytometric platelet-aggregate measurements.

### 2.10. Flow Cytometry

Samples were prepared in suspension by mixing platelets with 150 µL of PBS, incubating for 20 min in the presence of 0.5 µM Calcein AM. A Beckman Coulter Cytoflex flow cytometer (Beckman Coulter, Indianapolis, IN, USA) was used for the experiment. Readings were collected using the side scatter (SSC) and FITC data. SSC area versus height readings were plotted for data analysis. Cytoflex-generated FCS flow data files were analyzed using FlowJo 10.10 software (BD Biosciences, Ashland, OR, USA). X-axis (FITC-A): Measures green fluorescence intensity from calcein labeling (10^2^ to 10^7^). Y-axis (Count): Indicates the total cell/particle count (up to ~50–80 events per bin). Data are displayed in Results and Discussion Section 3.2.

### 2.11. Statistics

Categorical variables are reported as frequencies and percentage. Data presented are the mean ± SE from at least 4 independent platelet preparations. The distribution of data for continuous variables was evaluated with the Shapiro–Wilk test of normality and/or inspection of histograms. Statistical procedures were performed with Stata 14.0 (StatCorp, College Station, TX, USA) and GraphPad Prism 5.0 (GraphPad Software, La Jolla, CA, USA). *p*-values were two-sided.

To evaluate the effect of zinc chloride concentration (0, 1, and 2 mM) on the JAM-A D1 or JAM-C D1 circular dichroism ellipticity ratios (222:210 and 229:210), statistical significance was determined using a one-way Analysis of Variance (ANOVA) performed independently for each ratio dataset. If the ANOVA reveals a significant treatment effect, then *p* < 0.05.

## 3. Results and Discussion

**Figure 1:**
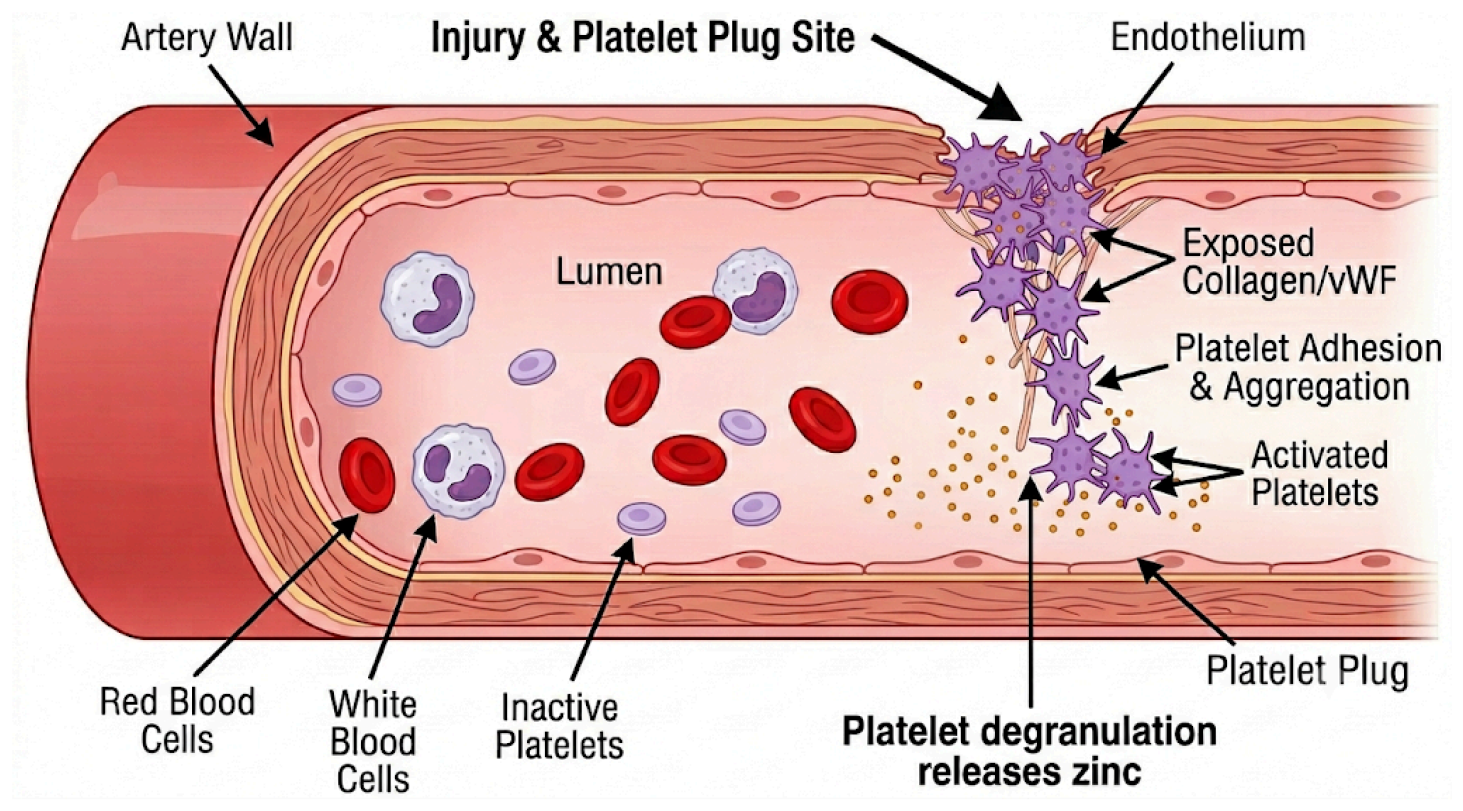
Contribution of degranulated platelets and Zn^2+^ in the regulation of hemostasis. Schematic representation of the events that follow injury in the endothelium. Platelets in circulation are exposed to endothelial factors (e.g., ADP) and initiate platelet plug formation at the site of injury. Local levels of Zn^2+^ are the result of degranulation of platelets. Zinc has been associated with platelet aggregation, coagulation, and fibrin clot formation [17].

Primary hemostasis is the rapid, initial response to vascular injury, in which platelets form a temporary plug to limit blood loss (Figure 1). Platelet degranulation occurs after activation, when platelets release the contents of their intracellular granules—such as ADP, calcium, zinc, and thromboxane A_2_—which amplify platelet activation and recruit additional platelets to the injury site. For a recent review, consider Scridon 2022 [28].

### 3.1. JAM Antibodies

Our first interest extended to the contributions of both JAM-A and JAM-C to platelet plug formation. Initially, we replicated Sobocka and colleagues’ work [13] and incubated platelets in the presence of antibodies against JAM-A and JAM-C. We examined shape shifting under control classical activation of platelets using ADP [29].

**Figure 2:**
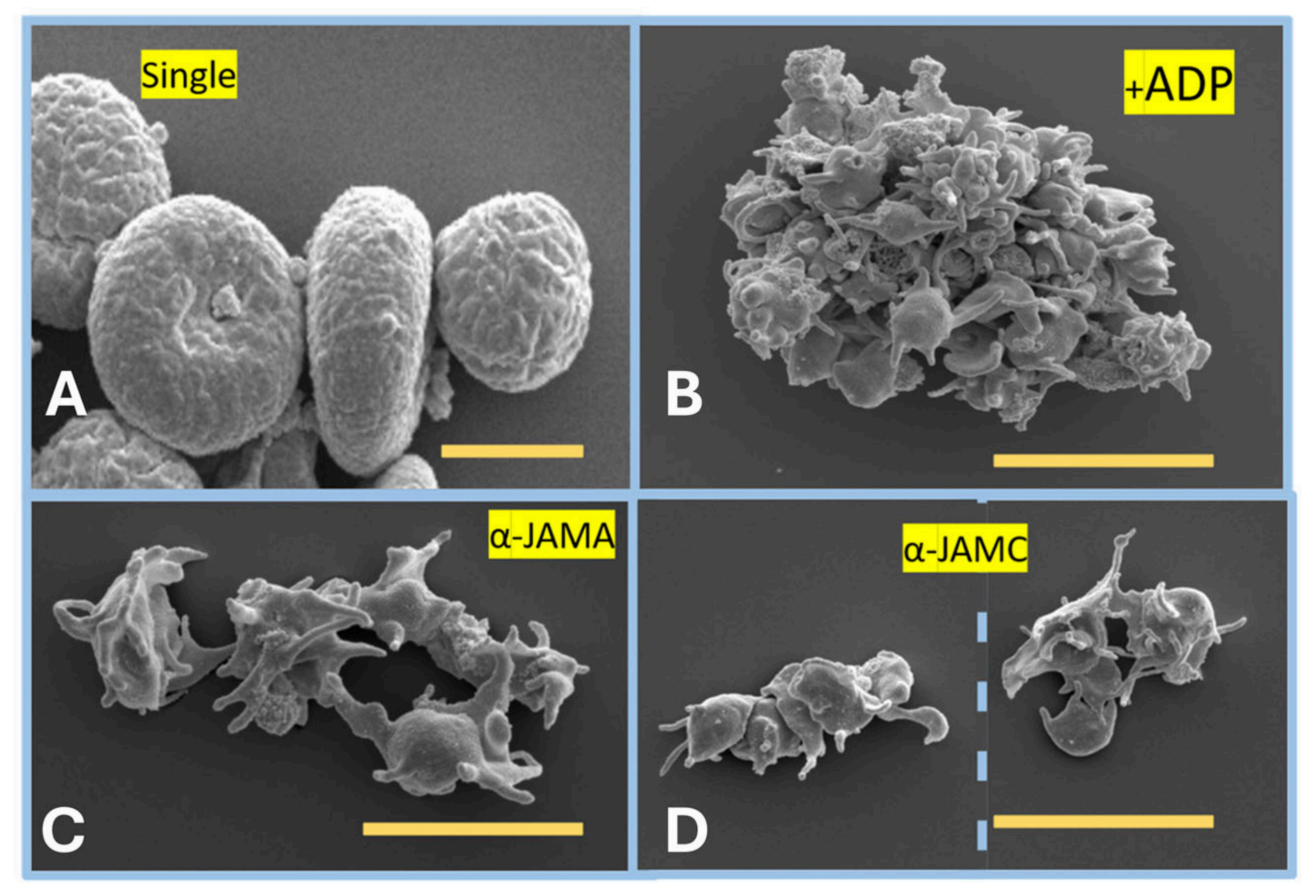
Star-shaped morphology of platelets. In this image, we present (**A**) inactive platelets (round); size bar is 2 µm. (**B**) Changes in morphology (star-shaped) and aggregation of platelets exposed to ADP; size bar is 10 µm. (**C**) Changes in morphology of platelets exposed to anti-JAM-A; size bar is 10 µm. (**D**) Anti-JAM-C; size bar is 10 µm. The blue dashed line separates two groups of platelets in the same image.

We observed (Figure 2) that under the influence of ADP, platelets started to aggregate, changing morphology to a star shape. Incubating platelets with antibodies for JAM-A or JAM-C triggered the change in morphology with only minimal aggregation. This agrees with previous reports by Sobocka and colleagues work [13]. Perhaps the lack of aggregation is due to JAMs being blocked by the antibody from engaging in cell adhesion. The morphology changes seem to indicate that antibody binding triggers intra-platelet changes in its cytoskeleton through JAMs’ intracellular domain [11]. Platelets contain a highly dynamic cytoskeleton composed of actin filaments and microtubules that maintain their discoid shape and facilitate rapid reorganization upon activation. Platelets express JAMs and some members of the zonula occludens (ZO) family, which are involved in signaling and shaping cells [30]. A possible artifact observed in Figure 2 is the appearance of single platelets. Standard centrifugation and processing steps for scanning electron microscopy can cause minor artifactual swelling or surface ruffling. These units are isolated single units completely lacking the extended, complex pseudopodia seen upon true activation (Figure 2).

### 3.2. Zinc and Platelet’s Aggregation

ADP is a key platelet activator in hemostasis: when vessels are injured, platelets release ADP, which then amplifies platelet activation and aggregation to build the hemostatic platelet plug [1,6]. ADP acts mainly through platelet purinergic receptors, especially P2Y1 and P2Y12, to trigger shape change, increase intracellular calcium, and suppress cAMP, all of which promote aggregation. This makes ADP a major positive-feedback signal that recruits more platelets to the injury site and stabilizes the clot [31]. Based on this information, we decided to test the ability of the components of our study in relation to platelet aggregation using Flow Cytometry. This technique is accepted in the field of platelet aggregation and hemostasis.

**Figure 3:**
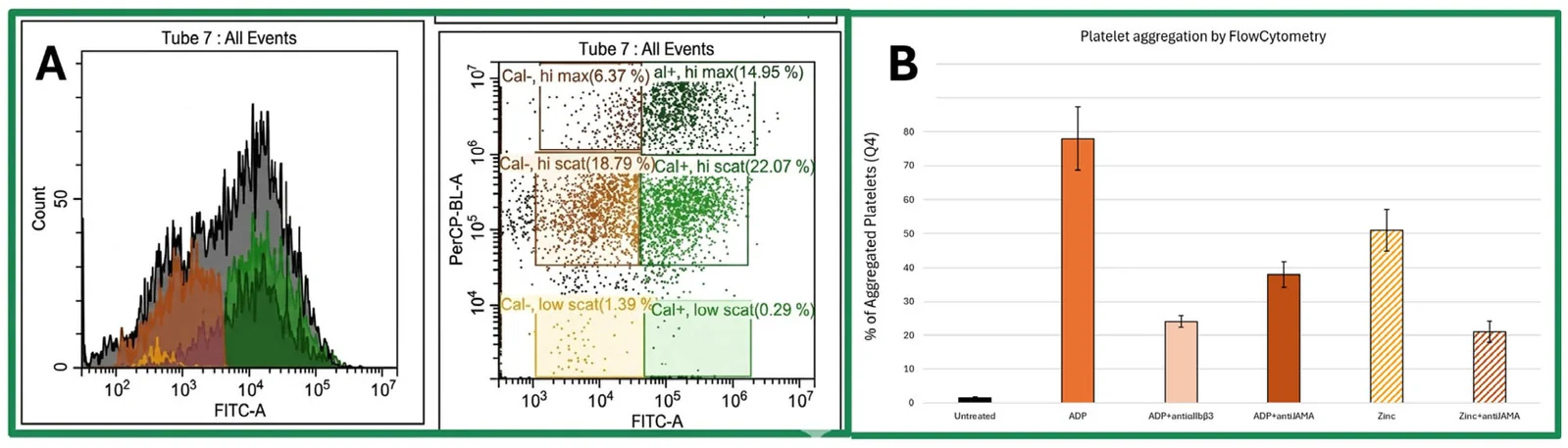
Flow Cytometry of platelets. (**A**) Typical data collected from the Flow Cytometer. Left panel X-axis (FITC-A): measures green fluorescence intensity from calcein labeling; Y-axis (Count): indicates the total cell/particle count. The right panel has the following axes: FITC-A (green fluorescence, horizontal) vs. PerCP-BL-A (vertical signal/scatter), and gates 6 quadrants to identify aggregation states of platelets. (**B**) Comparison of platelet aggregation levels by ADP and zinc. Reduction in platelet aggregation by anti-αIIbβ3 and anti-JAM-A after induction with ADP. Finally, reduction in platelet aggregation by anti-JAM-A after induction with zinc. All treatments are statistically different than the untreated platelets and ADP treatment (*p* < 0.01). Zinc treatment is statistically different than Zinc + anti-JAM-A (*p* < 0.05).

To isolate the results from the effects of antibody usage, we employed Calcein-AM to label viable platelets and track their aggregated states (Figure 3A) [32]. Figure 3A displays the data in two different ways: in the left panel, the X-axis (FITC-A) measures green fluorescence intensity from calcein labeling (10^2^ to 10^7^), while the Y-axis (Count) indicates the total cell/particle count (up to ~50–80 events per bin). The population distribution shows a broad distribution with two main shaded subpopulations overlaid in different colors: the orange region (low FITC-A) represents calcein-negative or low-fluorescence events; the green region (high FITC-A) represents calcein-positive fluorescent platelets. 

The right panel of Figure 3A has the following axes: FITC-A (green fluorescence, horizontal) vs. PerCP-BL-A (vertical signal/scatter). Gated Quadrants/Gates: We plot six distinct gates defined by calcein status: (Q1) Top-right green gate representing high-fluorescence, high-scatter events. In ADP-induced platelet aggregation, higher scatter and higher total fluorescence per event typically correspond to aggregated platelet clusters; (Q2) Middle-right green gate corresponding to single fluorescent platelets or small aggregates with moderate scatter; (Q3) Bottom-right green gate showing minimal low-scatter, positive-fluorescence debris or small fragments; (Q4) Middle-left orange gate showing non-fluorescent (unlabeled) platelets or particles with moderate scatter; (Q5) Top-left brown gate showing high-scatter, non-fluorescent aggregates or large background particles; (Q6) Bottom-left yellow gate representing background noise or low-scatter non-fluorescent debris. The presence of events in Q1 and Q5, the hi-max gate, indicates the formation of larger aggregates/complexes following ADP stimulation, as aggregated platelets produce a higher combined scatter and fluorescent signal per event. The presence of more than one type of aggregation of platelets has been described before. Activated platelets form in an all-or-nothing manner, which suggests that during activation, platelets commit to becoming procoagulant or non-coagulant and that there are differences in intracellular signaling between these two subpopulations [33]. Calcium is required for both activated and non-activated platelets for further activation; however, how the variation in calcium signaling may activate platelets also varies and is not simply an all-or-nothing outcome [33,34,35]. Additional images are presented in Supplementary Figure S1.

We observed with this strategy, as expected, that both ADP and Zinc aggregate platelets, with ADP aggregating platelets to a greater extent (Figure 3B). Prior to engaging platelets with antibodies, we referenced previously published methods that have used anti-αIIb or anti-β3 antibodies as positive controls for platelet aggregation [36] (Figure 3B). Having confirmed the effects of anti-integrin antibodies, we treated platelets with anti-JAM-A antibody. Anti-JAM-A can reduce aggregation levels induced by both ADP and Zinc (Figure 3B). Our data from the incubation with anti-JAM-A agree with Sobocka and colleagues’ work [13] and our own results presented here (Figure 1). Anti-JAM-A reduces aggregation, perhaps by reducing the availability of JAM-A to form self-interactions between platelets, preventing platelet–platelet interactions. We predict that ADP- treated platelets may have less aggregation but will display star-shaped platelets [13]. Zinc-activated platelet aggregation may not contain star-shaped platelets.

### 3.3. Does Zinc Interact with JAMs?

We have examined how zinc and JAMs influence platelet aggregation. Does zinc interact with JAMs’ extracellular soluble domains? JAM proteins have two immunoglobulin domains (D1 and D2) [11,37]. For that purpose, we examined how each extracellular domain of JAM-A and JAM-C behaves in solution. We prepared plasmids that expressed JAM-A N-terminal domains D1 and D2 close to the membrane. We repeated the strategy with JAM-C domains. Proteins are expressed and purified and then subjected to size-exclusion chromatography (SEC).

**Figure 4:**
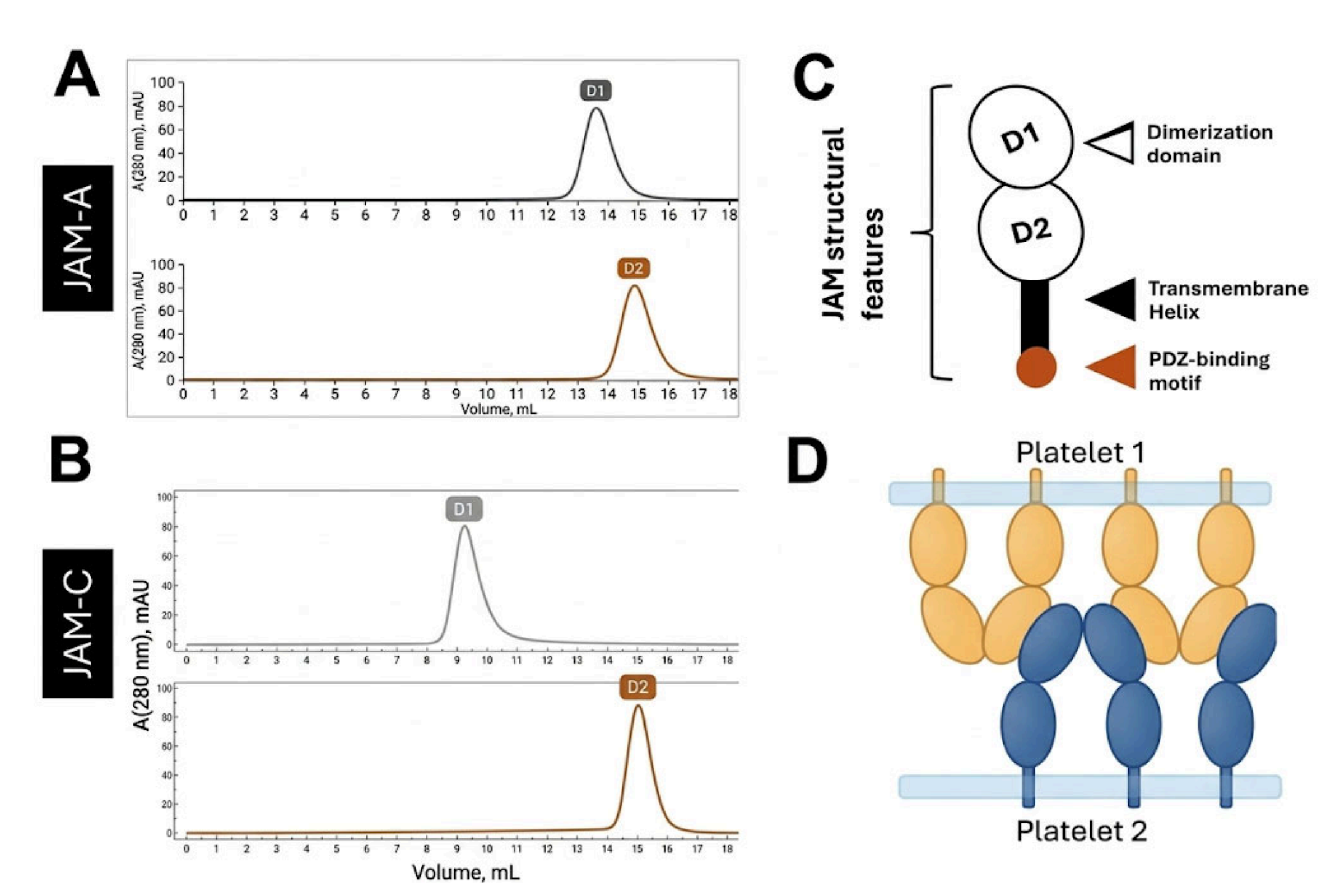
SEC of independent domains of JAM-A and JAM-C. (**A**) Chromatograms demonstrate that D1 in JAM-A is larger than its monomeric form (11 kDa), while D2 is monomeric (11 kDa). (**B**) Chromatograms of JAM-C domains indicate that D2 remains monomeric (11 kDa), while D2 is highly aggregated (above 600 kDa, close to the void volume, 9 mL). (**C**) Basic structures of a Junctional Adhesion Molecule (JAM). (**D**) We provide a model of active extracellular domains of JAMs interacting between platelets; the dimerization domains are responsible for platelet–platelet adhesion. Observed in this model is the concept that JAMs may interact in cis-interactions and also trans-interactions. The latter leads to cell-adhesion or platelet–platelet interactions. Vo corresponds to 9 mL.

Figure 4 indicates that the dimerization domain (D1) of JAM-A and JAM-C has a size beyond their monomeric sizes. We have presented similar results previously [20]. What has not been characterized is how oligomerization, to be dimeric or larger, is accomplished by the D1 domains of JAMs. Some evidence seems to indicate that cell-adhesion is associated with trans-configuration, while dimerization can occur where JAMs bind in cis-configuration [38] without resulting in cell-adhesion.

**Figure 5:**
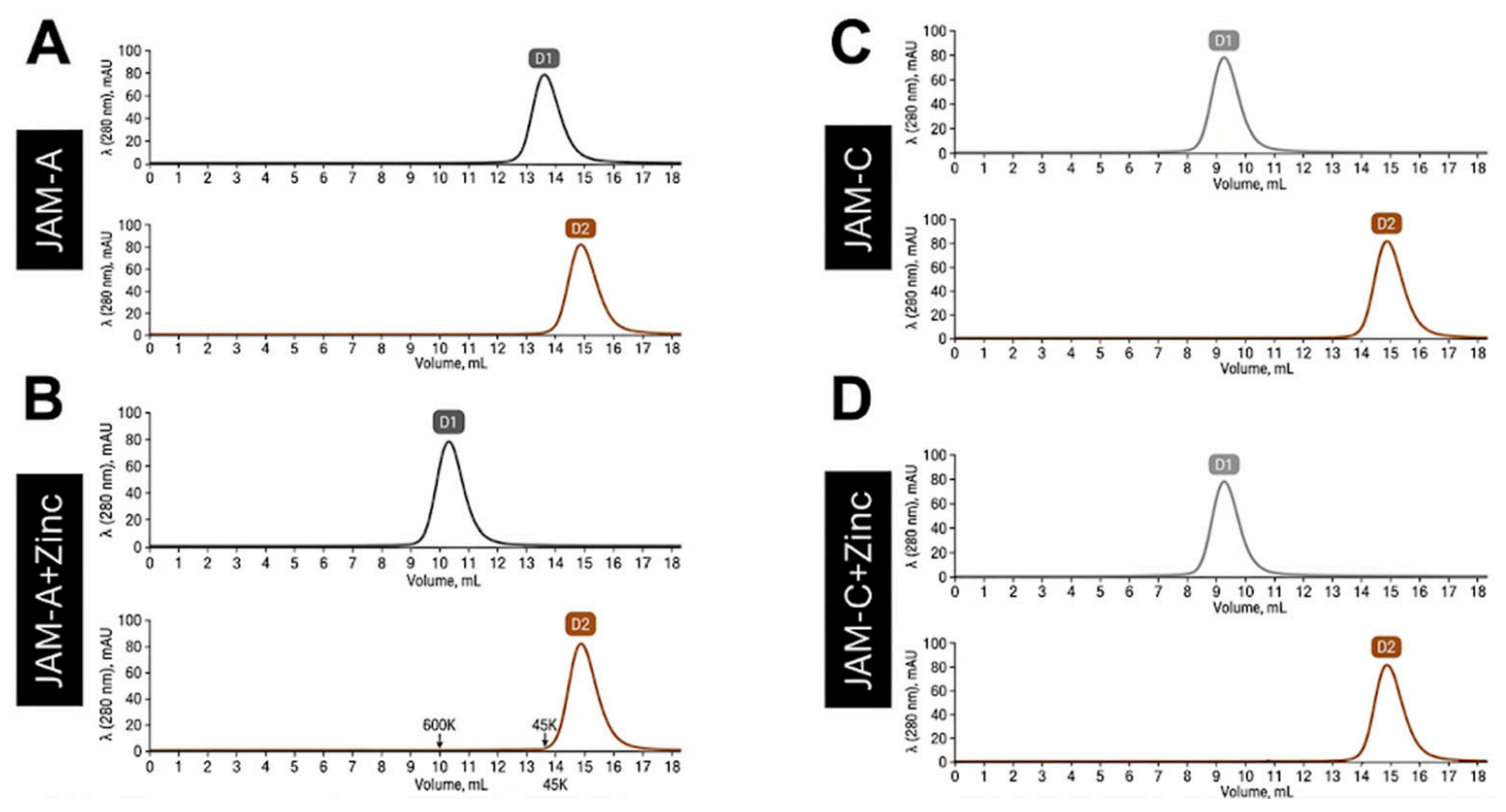
SEC of independent domains of JAM-A and JAM-C in the presence of Zinc. Chromatograms replicate the data presented in Figure 4 and expand the results of the different domains in the presence of zinc. For the control, we present data collected for the elution volumes of the protein standards: 600 kDa elutes around 10 mL, and the 45 kDa standard elutes at 13.5 mL. The void volume of this column is 9 mL. (**A**) Chromatogram of JAM-A domains in the absence of zinc. These are also presented in Figure 4. (**B**) Chromatogram of JAM-A domains in the presence of zinc. (**C**) Chromatogram of JAM-C domains in the absence of zinc. These are also presented in Figure 4. (**D**) JAM-C domains in the presence of zinc.

We proceeded to expose the different domains to zinc (2 mM ZnCl_2_) and repeated SEC experiments. Our findings are presented in Figure 5. It is observed in these chromatograms that D2 domains are not influenced by Zinc. On the other hand, D1 of JAM-A is largely aggregated, from the dimer observed (Figure 4A) to over 600 kDa in the presence of zinc. We could not confirm that JAM-C D1undergoes similar changes in its aggregation number. The dimerization domain of JAM-C is already at the maximum size detectable by SEC.

### 3.4. What Is the Effect of Zinc on JAM-A Soluble Domain?

**Figure 6:**
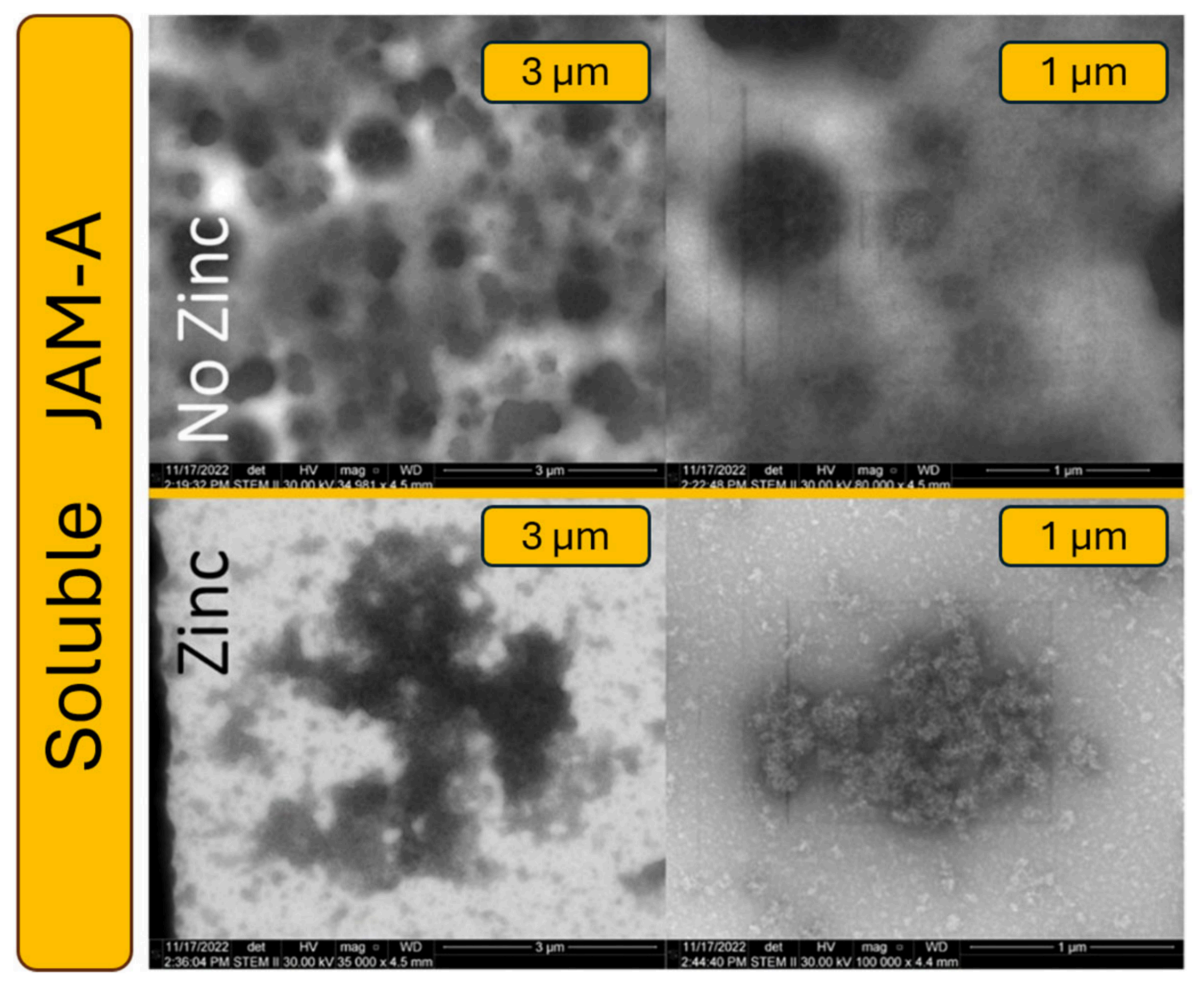
Electron microscopy imaging of purified extracellular soluble domain of JAM-A. For each condition (no Zinc, and with 1 mM ZnCl2), we present two different magnifications (3 and 1 µm).

Considering that JAM-C is largely aggregated in the absence of zinc, we estimated that the best approach to demonstrate JAM’s interactions with zinc could be using only JAM-A. Thus, we expressed and purified the extracellular domain of JAM-A, following previously published methods [20,24], and examined its organization using Electron Microscopy (EM), Figure 6.

Using EM imaging, we observe that the soluble extracellular domain of JAM-A (D1 and D2) appears globular. The structure observed for JAM-A in the presence of Zinc is an aggregate that does not appear globular. These observations support our point of view that Zinc largely influences platelet plug formation.

### 3.5. Circular Dichroism Spectroscopy

We decided to analyze the effects of Zinc on D1 of JAM-A and JAM-C using Circular Dichroism (CD) spectroscopy. We were not able to demonstrate that JAM-C D1 domains aggregated beyond the size observed in the absence of Zinc (Figure 5). We decided to test if zinc would have an effect on the secondary structure of the JAM-C dimerization domain, D1. CD spectroscopy is an excellent, rapid technique for analyzing the secondary structure (α-helices, β-sheets), folding, stability, and conformational changes in proteins and nucleic acids in solution. It is used in biophysics to monitor structural changes caused by mutations, temperature, pH, or ligand binding.

Spectra used signal ratios of 222 nm/210 nm and 229 nm/210 nm. Measuring 222 nm/210 nm and 229 nm/210 nm in far-UV CD can tell you about secondary-structure balance and helix packing/perturbation, not just total helicity. In practice, 222 nm is commonly used for α-helix content, while ~210 nm sits near the stronger backbone band where helical, disordered, and other conformational contributions can differ, so the ratios can be used as internal spectral descriptors rather than absolute structural readouts. This approach lets us account for the aggregation we observed in these proteins while also being able to estimate their secondary structure [39,40]. 

**Figure 7:**
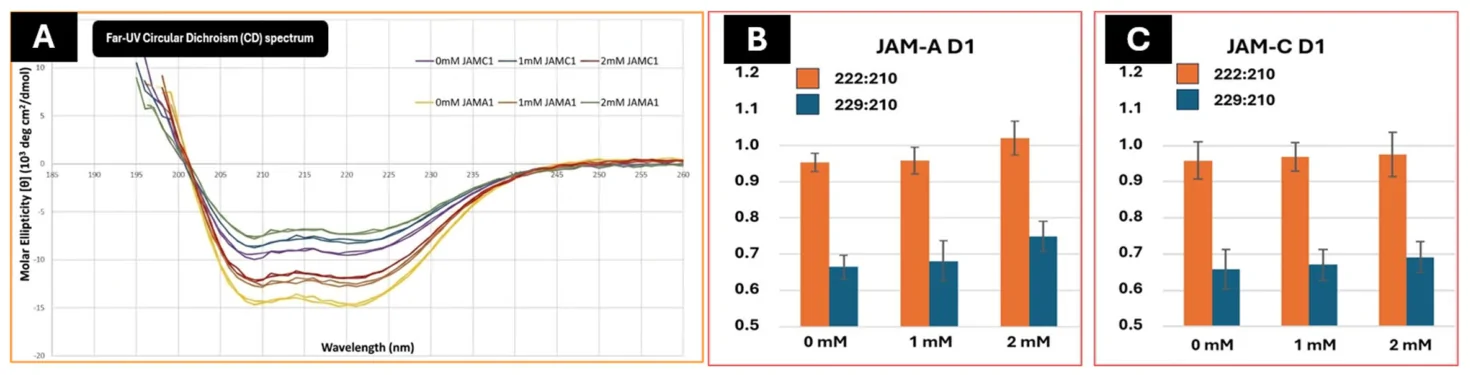
Far-UV Circular Dichroism. (**A**) Far-UV CD spectrum. Curves represent JAM-A domain 1 (JAMA1) and JAM-C domain 1 (JAMC1) in the presence of 0, 1, or 2 mM zinc chloride. (**B**,**C**) Circular Dichroism (CD) spectra using signal ratios of 222 nm/210 nm and 229 nm/210 nm of JAM-A D1 (*n* = 4) and JAM-C D1 (*n* = 3). Statistical analysis reveals a *p* < 0.05 for JAM-A D1 but no statistical difference for JAM-C D1.

Figure 7 contains the far-UV Circular Dichroism spectrum of both JAM-A and JAM-C domain 1 (D1). The data presented in Figure 7B,C analyze the ratio of signal at 222:210 and 229:210. In Figure 7B, JAM-A D1, we observed a rise in 222:210 toward ~1.0, suggesting that Zinc is promoting or stabilizing the α-helical structure of JAM-A1: the peptide becomes more helical or more ordered as Zn^2+^ increases. Rising 229:210 is particularly informative—this strongly suggests zinc is inducing helix packing, oligomerization, or an environmental change around aromatic residues, consistent with metal-coordinated helix-helix association or aggregation. Together, both ratios rise in parallel, pointing toward a zinc-driven conformational stabilization or self-association event rather than simply more helix content alone. Such biphasic stabilization followed by destabilization has been reported in other zinc-binding proteins [41].

For JAM-C D1, this domain maintains its secondary structure regardless of the concentration tested. There is no evidence of concentration-driven helix perturbation, aggregation, or conformational switching. The consistent gap between the two ratios (~0.30 units) across all conditions suggests the spectral shape is stable and reproducible. 

JAM-A D1 appears to be zinc-responsive, meaning its conformation or assembly state changes with zinc. This could reflect zinc coordination driving oligomerization, which is biologically relevant if JAM-A is designed to interact with lipid membranes or proteins in a zinc-dependent manner. The larger error bars at 2 mM may suggest heterogeneity in the Zn^2+^-induced state, possibly a mix of monomeric and oligomeric species.

Overall, these results show us that JAM-A D1 undergoes the clearest zinc-dependent conformational transition. This selective effect may reflect intrinsic differences in domain flexibility, as has been noted for other adhesion proteins such as cadherins under mechanical stress [42,43]. It also resonates with CD studies of intrinsically disordered proteins like SNAP-25, where environmental factors (lipids, ions) can shift structure in a graded manner [44]. CD analysis highlighted the protein (JAM-A) and domain-specificity of zinc. 

These findings support our findings from SEC studies (Figure 5) that D1 of JAM-A is the most structurally flexible domain, while D2 from JAM-A or JAM-C is not responsible for aggregation. 

### 3.6. Surface Plasmon Resonance

Surface Plasmon Resonance (SPR) was used to quantify the binding affinities of the D1 domains of both JAM-A and JAM-C under the effects of Zinc. We used 1 mM zinc supplementation for the conditions and were able to interpret different kinetic parameters of these interactions like association rate constant (ka), dissociation rate constant (kd), and equilibrium dissociation constant (KD). These values help us understand the strength and dynamics of homotypic and heterotypic interactions within JAM-A and JAM-C. 

**Figure 8:**
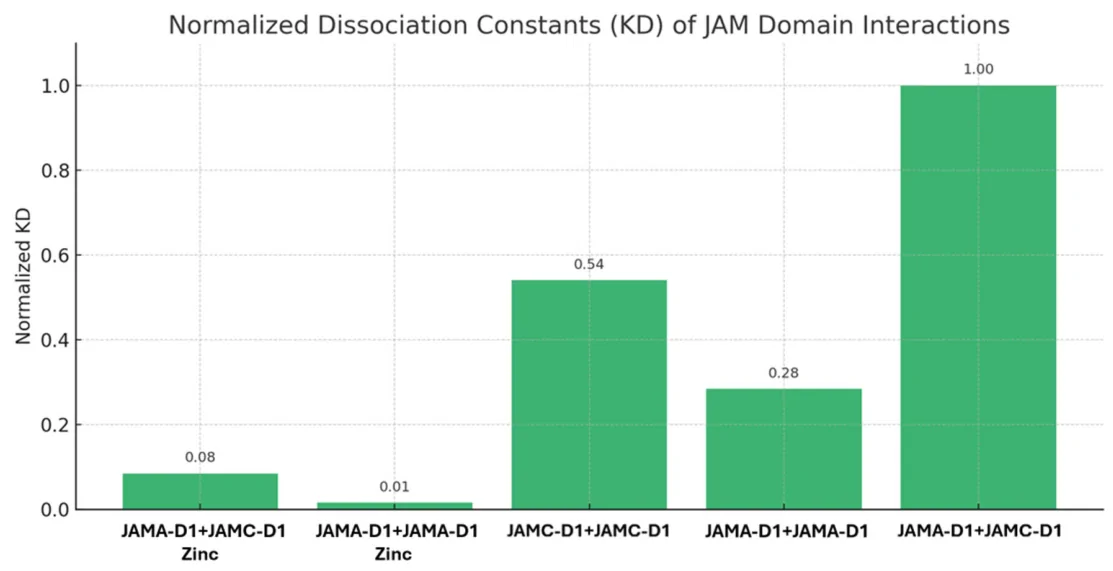
Normalized equilibrium dissociation constants (KD) of JAM-A and JAM-C interactions under zinc and zinc-free conditions. All results presented here were calculated from data that can be found in Supplementary Table S1, including sensograms (Supplementary Figure S2).

The results (Figure 8) show that the strongest interaction occurred between JAM-A D1 and JAM-A D1 in the presence of zinc, with a KD of 1.53 × 10^−6^ M. The weakest interaction was JAM-A D1 vs. JAM-C D1 in the absence of zinc, with a KD of 1.04 × 10^−4^ M. The difference in magnitude is of 2 orders, which seems to indicate that in the presence of zinc, JAM-A self-association and dimerization/oligomerization are more favorable. To visualize the differences in binding affinity, the KD values were normalized to the weakest interaction, which was set to 1, as shown in Figure 8. These results are consistent with previous studies showing Zn^2+^ acts as a regulatory cofactor that promotes JAM dimerization by altering conformation or charge distribution of the extracellular domains [21,24]. In the context of platelet aggregation, this supports a model in which zinc released during activation strengthens JAM-A-dependent interactions, contributing to inter-platelet adhesion and stabilization for a more efficient platelet plug. 

SPR analysis quantified the binding kinetics underlying these observations. JAM-A1 homodimers are bound ~20-fold more tightly in the presence of zinc compared to the control. By contrast, JAM-A1 vs. JAM-C1 interactions were weaker and less zinc-dependent, while JAM-C1 homodimers exhibited minimal zinc sensitivity. Mechanistically, zinc may promote JAM-A dimerization by neutralizing repulsive charges or stabilizing extracellular domain conformations, a phenomenon documented in other immunoglobulin superfamily members [7]. Together, CD, SEC, and SPR provide a convergent picture: zinc promotes JAM-A adhesion, which may result in more trans-interactions.

### 3.7. Designing a Peptidomimetic of JAM-A to Alter Platelet Aggregation

**Figure 9:**
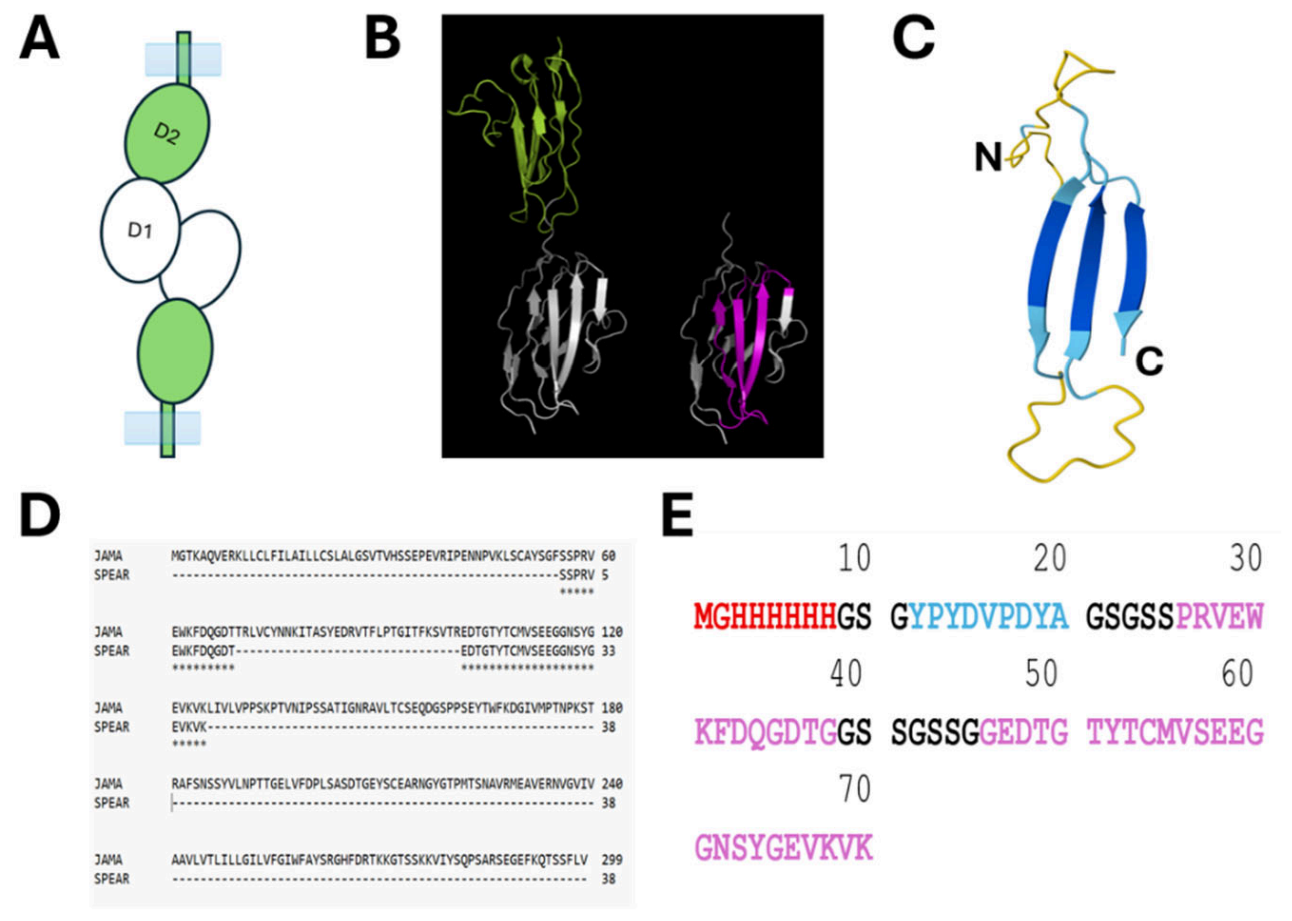
Design of JAM-A peptidomimetic, named SPEAR. (**A**) Model of JAM-A as a cell-adhesion molecule. Dimerization domain D1 (white), domain 2, D2 (green). (**B**) Crystal structure of the extracellular domain of JAM-A (PDB ID: 1nbq [46]) showing D1 (white) and D2 (green). D1 with the SPEAR highlighted in magenta. (**C**) SPEAR and its predicted structure (alphafoldserver.com, 05/14/2026); the blue structure is predicted with over 90% confidence, and the yellow structures are predicted with less than 70% confidence. (**D**) ClustalW (05/14/2026) alignment of JAM-A (accession number: Q9Y624) and SPEAR. (**E**) Amino acid sequence of SPEAR: 6xHis tag (red), HA tag (blue), JAM-A sequence selected to bind zinc (magenta). In black are amino acids acting as linkers.

Our data collected here suggest that zinc associates with JAM-A via its D1 domain. We have presented evidence using imaging, purified proteins, CD, and SPR. Focusing on D1 of JAM-A, we analyzed its amino acid sequence and crystal structure (Figure 9). We designed a small peptide derived from this D1 domain where the surface selected contains the greatest concentration of negatively charged amino acids, as described in the literature, to be zinc-binders [45].

Figure 9 contains the basic strategy employed to engineer a peptidomimetic based on the JAM-A dimerization domain (D1). We named the peptidomimetic SPEAR. The prediction of the folding of the amino acid sequence selected (Figure 9C) seems to indicate with high probability that the amino acids will retain their β-sheet properties and form a flat surface. 

**Figure 10:**
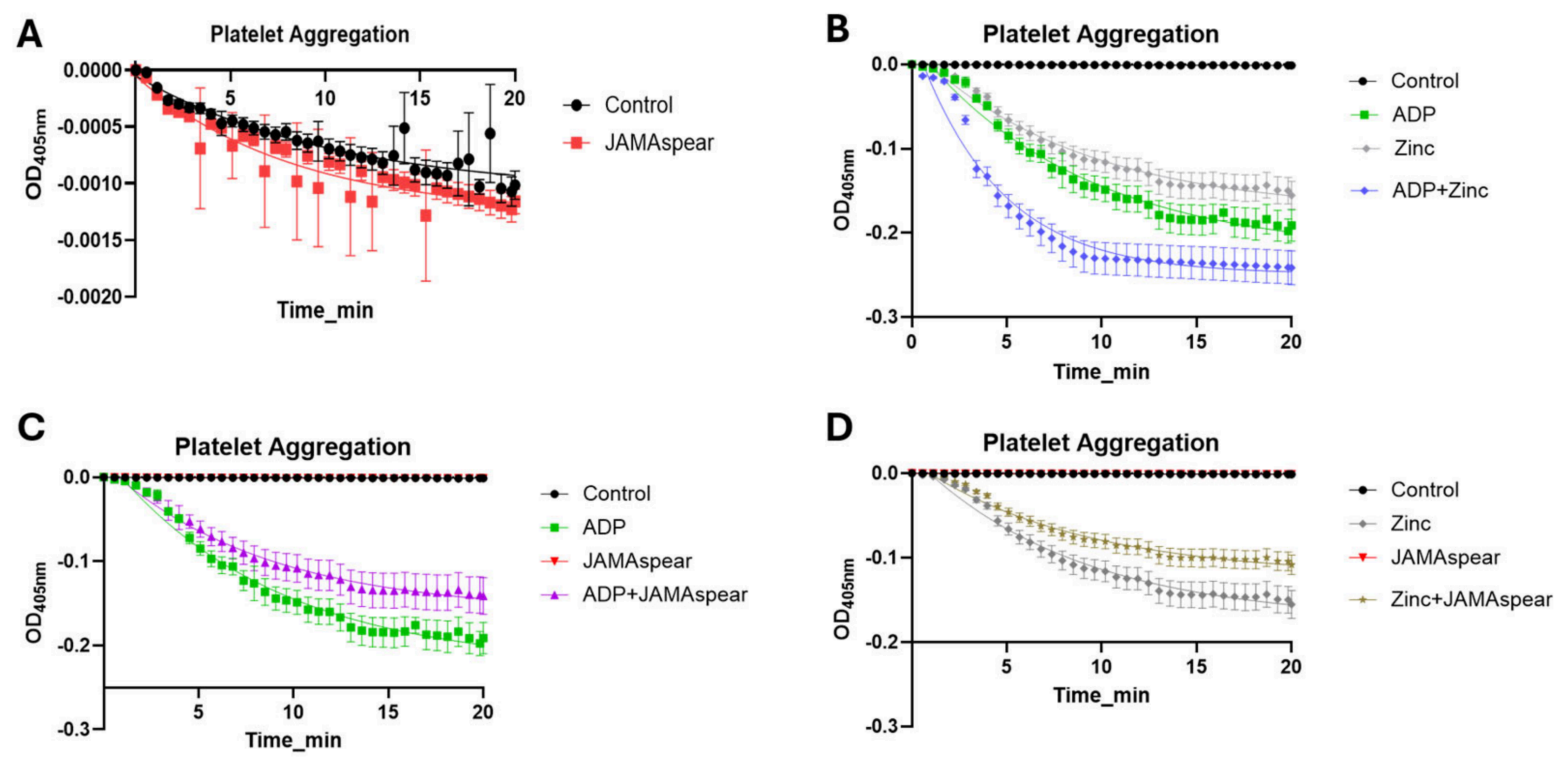
Platelet aggregation measured at OD_405nm_. (**A**) PRP in the presence of PBS (Control) or JAMAspear. (**B**) PRP in the presence of PBS (Control), ADP, zinc, and a mixture of ADP and zinc. (**C**) PRP in the presence of PBS (Control), ADP, JAMAspear, and a mixture of ADP + JAMAspear. (**D**) PRP in the presence of PBS (Control), zinc, JAMAspear, and a mixture of zinc + JAMAspear. PRP is used in these experiments, and its aggregation is monitored at 405 nm for a period of 20 min. Reading each well at 405 nm every 20 s allows us to observe the dynamic aggregation of platelets under different conditions. Control (PBS) or 1 mM SPEAR did not change platelet aggregation compared to ADP or zinc treatments (panel (**A**)).

Continuing with the search toward the role of zinc in Primary hemostasis and platelet plug formation, we now have a tool (SPEAR) that could bind zinc and reduce its effects on platelets and platelet plug formation. We assessed platelet aggregation using a 96-well microtiter plate–based optical aggregometry approach, in which platelet aggregation is quantified from the decrease in absorbance accompanying increased light transmission through the platelet suspension [47]. Microplate-based light-transmission aggregometry has been independently optimized and validated for testing platelet aggregation across multiple agonists and conditions [48]. The primary reason for selecting 405 nm is that it sits in the visible light spectrum, just outside the range where proteins and nucleic acids naturally absorb light. The OD_405nm_ signal was used as an optical readout of the change in turbidity/light transmission that accompanies platelet aggregate formation. The selected wavelength was applied as part of the established microplate aggregometry workflow; it should not be interpreted as demonstrating absence of direct optical contributions from added zinc, copper, protein, or other sample constituents [47]. We exposed purified platelets (platelet-rich plasma, PRP) to ADP, zinc, and ADP + zinc without and with 1 mM SPEAR (Figure 10). PRPs in the presence of SPEAR alone (in the absence of an agonist like ADP or Zinc) do not trigger or alter the resting platelet state (Figure 10A), whereas it does inhibit agonist-stimulated aggregation (Figure 10C,D). The data collected are represented in Figure 10. Ranking the levels of aggregation through the plate reader at 405 nm, we observed that ADP + zinc > ADP > zinc caused platelet aggregation. The presence of SPEAR reduces platelet aggregation but does not eliminate it completely (Figure 10C,D). The increased aggregation when platelets were exposed to both ADP and SPEAR (Figure 10B) seems to highlight that platelet plug consolidation relies on cooperative, multi-receptor networks. ADP stimulation drives primary activation and initial degranulation, which releases internal zinc into the microenvironment. This endogenous zinc then binds to surface JAM-A to stabilize and sustain the late-stage aggregation process. Because SPEAR sequesters this released zinc, it blunts the downstream JAM-A component of the total ADP response.

Through the experimental data collected, we have presented evidence that indicates that there is a relationship between high levels of zinc (greater than 100 µM) and the protein JAM-A. JAM-A is present on the surface of platelets, where its functions of cell adhesion and cytoskeleton dynamics activation are inhibited [7]. Primary hemostasis triggers platelet degranulation that in turn releases zinc. Circulating plasma zinc is homeostatically regulated at approximately 10–15 µM, although the freely available/labile fraction is substantially lower because most plasma zinc is protein-bound [49,50]. We propose that the events that release zinc from platelets may increase the local concentration of zinc to levels that can trigger changes in surface proteins of the platelets, namely JAM-A. We have dissected the structural domains of JAM-A that are responsible for binding zinc and self-organizing, perhaps into both cis- and trans-conformations. This interaction, through its dimerization domain (D1), seems to induce platelet aggregation. When monitored at 405 nm, platelets aggregate to a lesser extent than with ADP, but ADP + zinc aggregate platelets beyond the effects of ADP alone. The use of peptidomimetics based on JAM-A D1, SPEAR, reduced the effects of zinc, ADP, and ADP + zinc on platelet aggregation.

**Figure 11:**
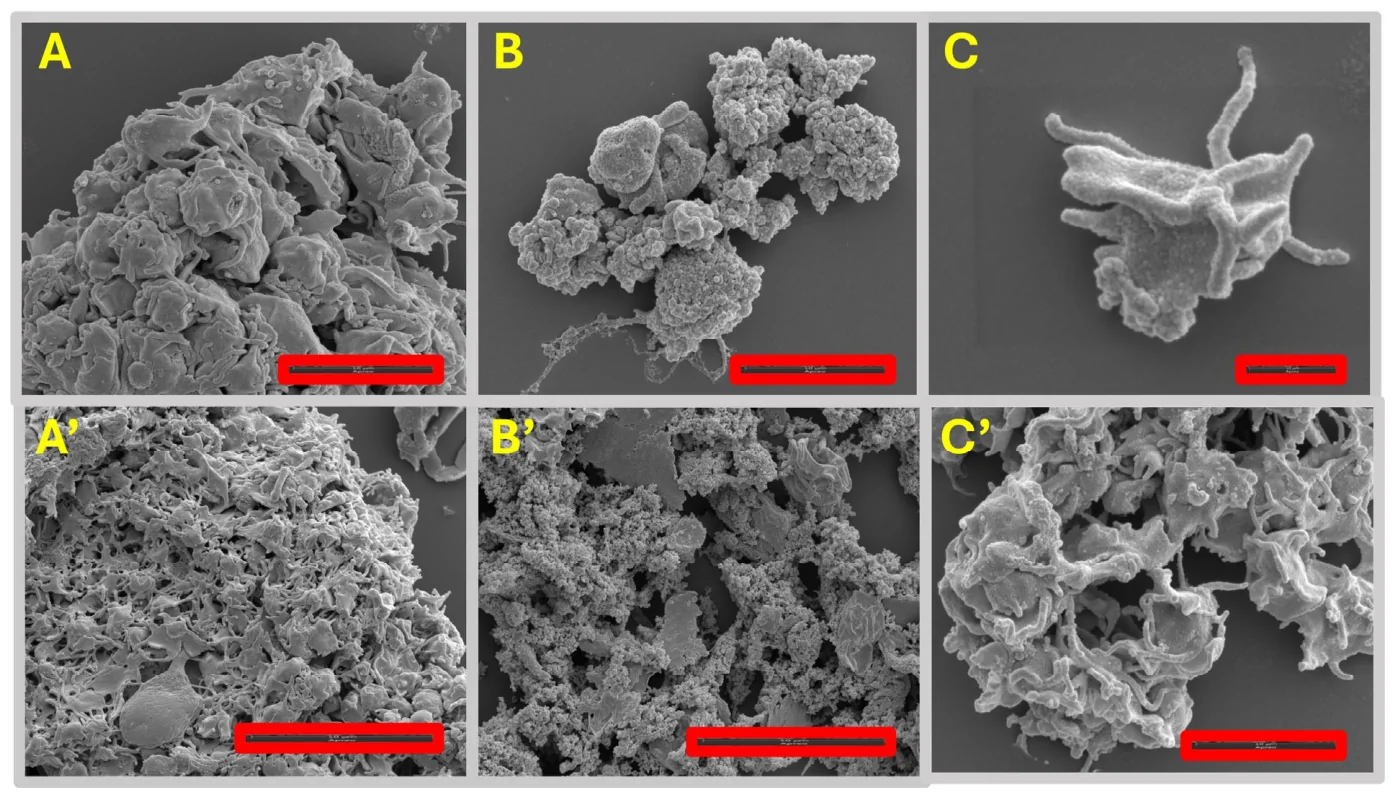
Electron microscopy images of platelets. (**A**,**A’**) Platelets exposed to ADP. Panel (**A**) size bar is 5 µm; (**A’**) is 10 µm. (**B**,**B’**) Platelets exposed to zinc. Panel (**B**) size bar is 5 µm, and (**B’**) is 10 µm. (**C**,**C’**) Platelets exposed to anti-JAM-A antibody. Panel (**C**) size bar is 1 µm, and (**C’**) is 5 µm.

Finally, we examined the role of zinc and JAM-A’s interaction in the formation of platelet plug. We presented evidence that zinc interacts with JAM-A, and that zinc can induce JAM-A oligomerization and platelet aggregation. We have reproduced experiments where platelets change morphology when exposed to anti-JAM-A antibody but do not induce large aggregation. JAM-A activation could be associated with platelet–platelet adhesion and with the cytoskeleton dynamics [38]. One question remains: does zinc activate both functions of JAM-A? We prepared images of platelets in the presence of ADP (control) and zinc (Figure 11).

We observed that the EM images (Figure 11) seem to indicate that, compared to ADP, where platelet aggregation shows star-shaped platelets, zinc shows platelet aggregates that appear less tight, and platelets do not have the classical morphology in the platelet plug (star shape). A similar pattern of zinc-induced platelet aggregation has been reported [51]. From these images, we conclude that zinc-JAM-A interaction seems to alter the adhesion of platelets but appears disconnected from the cytoskeleton dynamics. Additionally, in the image selected for Zinc aggregation (mid-panel in Figure 11), other artifacts are observed beyond the platelet’s aggregation. In the preparation of the platelets, treatment and staining, no thrombin/fibrinogen components were added to the purified system, meaning the fibers represent extensive, loose chains of structurally altered platelets rather than a potential fibrin mesh. Future studies of JAM-A in our laboratory will focus on the inactivation of JAM-A and JAM-C on the surface of platelets, the mechanism by which they can interact with each other or other partners like CD9, as suggested by the literature [13,52]. Other options may include the fibrinogen receptor [51].

## 4. Conclusions

We have presented evidence for the role of zinc in platelet aggregation, leading to platelet plug formation. We have shed light on the effects of zinc interacting directly with D1 of JAM-A, and perhaps D1 of JAM-C. Nevertheless, to distinguish the effects of zinc on JAM-C may require a different set of experimental tools and strategies. We have engineered a small peptide, based on JAM-A D1 (SPEAR). This peptide can interact with zinc and decreases platelet aggregation due to ADP or Zinc. We have also identified contrasts between ADP and Zinc aggregation. ADP signals through purinergic receptors, activates GPs, and degranulation. These events lead to platelet plug formation. Zinc, on the other hand, appears to only cause aggregation, perhaps via JAM-A or other surface proteins, but does not activate platelet shape change to increase the efficiency of platelet plug formation. Understanding the cellular mechanisms of platelet activation and their pharmacologic modulation is of major interest for basic and clinical research. Our findings lead us one step closer to deconstructing new aspects of primary hemostasis.

## Data Availability

Authors will make any materials (e.g., Spear) or data used to generate the graphs available upon request. Please direct requests to Dr. Mizrachi (dario.mizrachi@noorda.edu).

## Supplementary Materials

The following supporting information can be downloaded at: https://www.mdpi.com/article/10.3390/medicines13040028/s1, Supplementary Figure S1. FlowCytometry of Platelet Aggregation; Supplementary Figure S2. Surface Plasmon Resonance, Sensograms; Supplementary Table S1. Surface Plasmon Resonance calculated constants.
