# Supplementary material for: The Role of Zinc and Junctional Adhesion Molecule A Protein in Platelet Plug Formation
Source: Medicines (Basel). 2026 Sep 29;13(4):28. doi: 10.3390/medicines13040028 (PMC13692491; doi:10.3390/medicines13040028)
Supplement: Supplementary file 1 [file medicines-13-00028-s001.zip › medicines-4349003-supplementary.pdf]

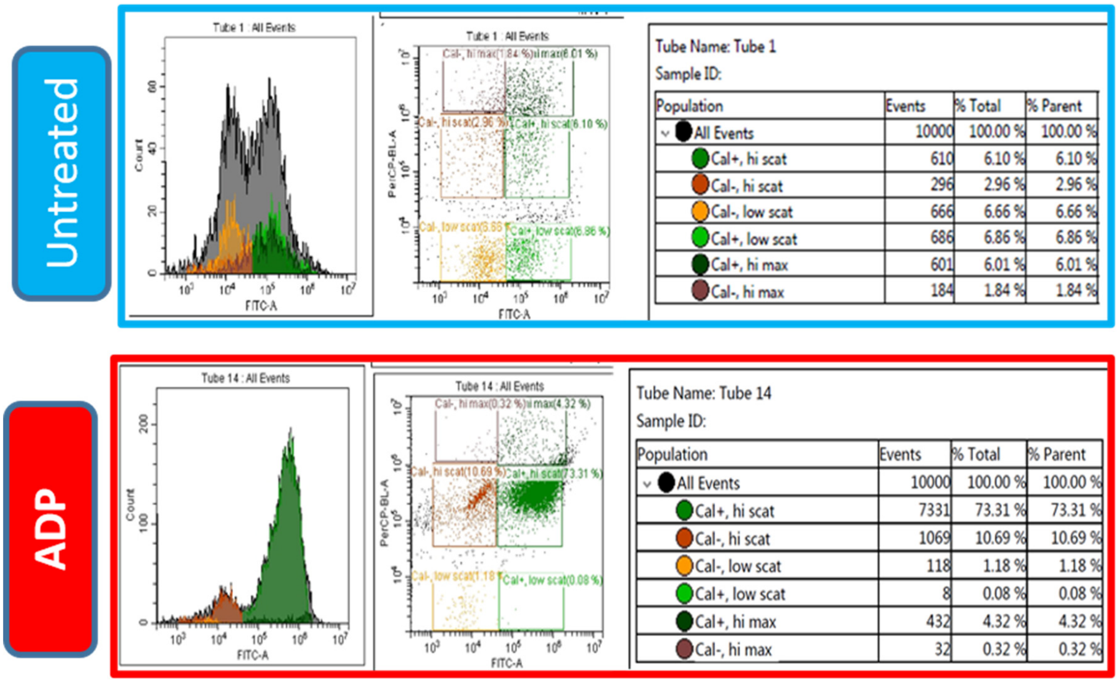

Figure S1. FlowCytometry of Platelet Aggregation. Here is data collected for platelet aggregation in FlowCytometry. Platelets are labeled green (FITC tracking) using Calcein-AM. Platelets are incubated with Calcein-Am and treatment (proteins, antibodies, ADP, etc.) for 20 minutes before injecting the sample in the Flow Cytometer. These two graphs show how cells are detected and further gated to identify single platelets (orange) and aggregated platelets (green).

Table S1. Surface Plasmon Resonance results for JAM-A and JAM-C proteins corresponding D1domains in the presence or absence of Zinc.

PPI Evaluated	Ka (1/(M*s))	Kd (1/s)	KD (M)
E-CAD vs. E-CAD *	2.23 × 103 ± 0.61 × 102	9.44 × 10-4 ± 8.88 × 10-5	7.67 × 10-7 ± 2.05 × 10-8
JAM-A vs. JAM-A	1.08 × 104 ± 7.10 × 103	6.48 × 10-4 ± 6.28 × 10-5	5.94 × 10-10 ± 5.52 × 10-9
JAM-C vs. JAM-C	9.55 × 104 ± 1.35 × 103	1.10 × 10-5 ± 3.69 × 10-6	1.15 × 10-10 ± 1.91 × 10-9
JAM4 vs. JAM4	1.37 × 105 ± 4.20 × 104	2.99 × 10-6 ± 6.24 × 10-7	2.19 × 10-11 ± 2.09 × 10-12
JAM-A vs. JAM-C	3.93 × 104 ± 7.71 × 103	8.53 × 10-5 ± 8.11 × 10-6	2.13 × 10-9 ± 3.99 × 10-10
JAM-B vs. JAM-C	2.41× 104 ± 2.77 × 103	4.11 × 10-5 ± 5.40 × 10-6	1.71 × 10-10 ±3.25 × 10-11

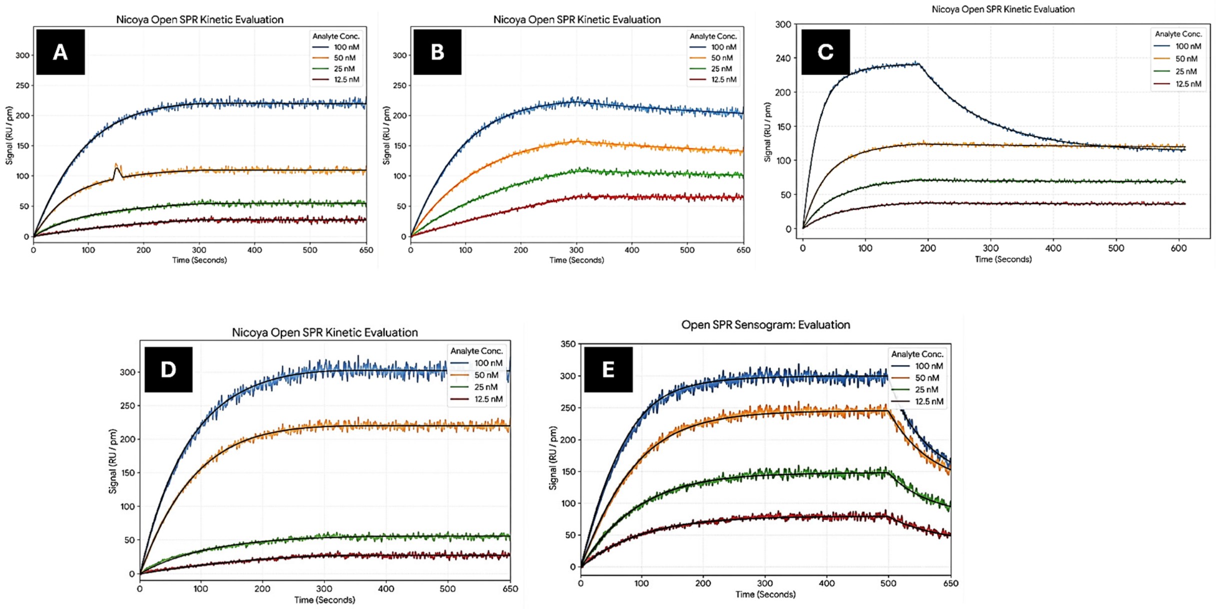

Figure S2. Sensograms obtained for each independent experiment. A) JAM-A D1 vD1 ; B) JAM-C D1 v D1; C) JAM-A D1 v D1 and zinc ; D) JAM-A D1 v JAM-C D1 ; E) JAM-C D1 v D1 and zinc.
